# Investigating Relationships between Balance Confidence and Balance Ability in Older Adults

**DOI:** 10.1155/2021/3214366

**Published:** 2021-11-26

**Authors:** Lara A. Thompson, Mehdi Badache, Joao Augusto Renno Brusamolin, Marzieh Savadkoohi, Jelani Guise, Gabriel Velluto de Paiva, Pius Suh, Pablo Sanchez Guerrero, Devdas Shetty

**Affiliations:** ^1^Biomedical Engineering Program, Department of Mechanical Engineering, School of Engineering and Applied Sciences, University of the District of Columbia, 4200 Connecticut Ave. NW, Washington, DC 20008, USA; ^2^Department of Mechanical Engineering, School of Engineering and Applied Sciences, University of the District of Columbia, 4200 Connecticut Ave. NW, Washington, DC 20008, USA; ^3^Department of Electrical and Computer Engineering, School of Engineering and Applied Sciences, University of the District of Columbia, 4200 Connecticut Ave. NW, Washington, DC 20008, USA; ^4^School of Engineering and Applied Sciences, University of the District of Columbia, 4200 Connecticut Ave. NW, Washington, DC 20008, USA

## Abstract

Increasing balance confidence in older individuals is important towards improving their quality of life and reducing activity avoidance. Here, we investigated if balance confidence (perceived ability) and balance performance (ability) in older adults were related to one another and would improve after balance training. The relationship of balance confidence in conjunction with balance performance for varied conditions (such as limiting vision, modifying somatosensory cues, and also base of support) was explored. We sought to determine if balance confidence and ability, as well as their relationship, could change after several weeks of training. Twenty-seven healthy participants were trained for several weeks during standing and walking exercises. In addition, seven participants with a higher risk of imbalance leading to falls (survivors of stroke) were also trained. Prior to and after training, balance ability and confidence were assessed via the Balance Error Scoring System (BESS) and Activities Specific Balance Confidence (ABC) Scale, respectively. Both groups showed improvements in balance abilities (i.e., BESS errors significantly decreased after training). Balance confidence was significantly higher in the healthy group than in the stroke group; however, ABC results reflected that balance confidence did not significantly increase after training for each. The correlations between balance ability and balance confidence were explored. Encouragingly, healthy participants displayed a negative correlation between BESS errors and ABC (i.e., enhancements in balance confidence (increases in ABC Scale results) were related to improvements in balance ability (decreases in BESS errors)). For the stroke participants, despite improvements in balance ability, our results showed that there was no relation to balance confidence (i.e., no correlation between BESS errors and ABC) in this group.

## 1. Introduction

With increases in average life expectancy, the importance of improving and maintaining balance has significant societal relevance for the aging population [1]. Falls are a major concern for all adults over 65 years old, and furthermore, stroke is considered the largest risk factor for falls [2–4]. But, aside from the act of falling, there is an overconcern about the anticipation of falling; this can ultimately limit one's confidence and willingness to go about their daily activities, thereby restricting their everyday quality of life [5–7].

Since the 1980s, the term fear of falling (FOF), defined as a persisting overconcern and anxiety regarding falls, had emerged and gained recognition as a health problem in aging individuals [8]. At first, FOF was thought to be a consequence of falling and was reported to range from 29 to 92% [8] in older adults that had previously fallen [9–12]. However, interestingly, FOF exists even in individuals that have not previously fallen (i.e., FOF symptoms may exist regardless of any previous physical trauma) [5, 6]. In older individuals, the FOF evolves from relationships tied to multifactorial influences (e.g., physical, psychological, and functional) [6].

Balance confidence and FOF are related, but distinct. Balance confidence is defined as an individual's confidence in their ability to maintain their balance while performing various activities [12, 13]. Previous research had established an association between falls and balance confidence in survivors of stroke [14]. Several studies had confirmed that both FOF and balance confidence are associated with poorer health status, functional decline, depression and anxiety, avoidance behavior, and decreased quality of life in older adults [1, 9, 15]. Although older individuals could be afraid to fall in general, survivors of stroke will have a range of neurological injury and deficits. These could lead to an enhanced loss of balance confidence and FOF. Stroke may cause motor control problems (e.g., paralysis or problems controlling movement and issues tied to balance and posture), sensory disturbances (e.g., loss of the ability to feel touch, sense how the body is positioned, pain, and numbness), problems understanding language, and issues tied to thinking and memory.

Currently, there exist several common measures tied to assessing falls and/or balance confidence. Some example assessments are as follows: Falls Efficacy Scale (FES), Activities Specific Balance Confidence (ABC) scale, and survey of activities and fear of falling in elderly (SAFE). The FES was created by Tinetti et al. [8] to examine fall-related self-efficacy, or self-confidence, in one's ability to avoid falling while doing home-based activities. The FES is based on a 10-question survey, with each question on a scale of 1 to 10, and the total score can range from 0 to 100. The ABC Scale was created by Powell and Meyers [13] for older adults that have higher functionality, but under the same premise/ideas related to FES. The 16-item survey includes activities performed outside of the home (greater difficulty than the FES), and one rates their confidence from 0% (no confidence) to 100% (completely confident). According to a study on balance confidence and FOF in older adults, the ABC Scale was found to be the strongest predictor of falls [16]. Furthermore, balance confidence quantified using the ABC Scale [13] has shown good internal consistency and test-retest reliability in, for example, individuals with chronic stroke [17, 18]. The SAFE was developed to assess the FOF and 11 activities of daily living; this measure is tied to assessing activity restriction or decreased quality of life.

An assessment that is commonly used, rapid, inexpensive, easy to perform (minimal (no) equipment needed), and helpful for quick monitoring and tracking of standing balance is the clinically used Balance Error Scoring System (BESS) assessment. It is well known that visual, somatosensory, and vestibular systems contribute to one's ability to maintain balance, as well as base of support (BOS), for example, the projected area between one's feet. Importantly, the BESS assessment allows for variation of somatosensory cues (i.e., hard vs. foam support surface) as well as variation of base of support, BOS, (stance widths of wide, tandem, and single-leg) while the participant has no visual cues (eyes are closed), thereby allowing for measured differences in balance (in terms of BESS errors) with increases in task difficulty. In terms of reliability of the BESS results, other studies have shown reliability is good [19–29]. Other commonly used functional balance tests (such as the Berg Balance Scale (BBS) and Timed Up and Go (TUG) Test), which also require minimal equipment, do not have the variation in task difficulty that BESS possesses. Furthermore, center-of-pressure displacement (via force plate measurements) is another metric to assess standing balance.

The aim of this study was to investigate balance confidence (perceived ability) and balance performance (ability) of the mature participants before and after training. Here, we test the general hypothesis that balance ability/performance is related to balance confidence in older adults. We examined differences in (1) balance confidence, (2) balance ability, and (3) the relationship (correlation) between balance confidence and balance ability before and after training.

## 2. Methods

All study activities were conducted within the Center for Biomechanical and Rehabilitation Engineering (CBRE) laboratory at the University of the District of Columbia, the protocol was approved by the Institutional Review Board (979744-1), and all participants gave their informed consent prior to participating in the study. Participants were mainly recruited via flyer postings around the University campus, as well as by word of mouth. Furthermore, the University of the District of Columbia's Institute of Gerontology disseminated flyers and information to prospective participants. We investigated balance ability and confidence predominantly in healthy older individuals; however, we also included a subset of older individuals within a group particularly prone to falls (survivors of stroke).

Thirty healthy and eight stroke participants (targeting 60–85 years old) enrolled in this study. Inclusion criteria included a score greater than 25 on the Mini-Mental State Examination to exclude those with possible dementia (all participants scored >28) and the inability to ambulate. Furthermore, inclusion criteria for survivors of stroke were that they were at least 6 months after stroke and able to walk without external assistance for at least 15 meters. One stroke participant and three healthy participants withdrew from the study. Thus, the results are presented for 7 stroke (66.1 ± 8.6 years old) and 27 healthy participants (69.6 ± 5.5 years old). Participant demographics are shown in Table 1 (survivors of stroke) and Table 2 (healthy participants).

### 2.1. Training Protocol

Participants underwent a 6-week exercise routine which consisted of two, 30-minute sessions/week. For these sessions, each participant was worked with individually within the CBRE laboratory. Over the course of several weeks, training progressively increased in difficulty. As stated above, it is well known that the visual, somatosensory, and vestibular systems affect balance; the training modified vision to make conditions more or less challenging (eyes-closed/eyes-open, respectively), as well as supported surface somatosensory cues (hard surface or foam surfaces). Thus, we utilized exercises, during several weeks of training, which required healthy and stroke participants to make use of diverse sensory information while attempting to maintain their balance. Base of support (BOS) was also modified between large and small BOS (e.g., double-leg, tandem, and single-leg stances) to increase task difficulty; a larger BOS allows for greater stability, while a smaller one leads to lesser stability. The training involved eyes-open/closed activities for walking exercises (forward and backward for wide and tandem stepping and side-stepping); foam exercises (standing, isolated leg exercises, squats and walking on hard surface, dense foam, or thick compliant foam); and walking over obstacles and more foam exercises [19]. During the sessions, the participants worked with the principal investigator and trainers (research assistants) which also served as spotters.

### 2.2. Assessment Protocol

Each participant served as their own control in those baseline (before training) assessments was compared to final (after training) assessments. In order to assess participant balance, assessments before and after training (i.e., Balance Error Scoring System (BESS) and Activities Specific Balance Confidence (ABC) scale) were conducted. The BESS assessment was used to measure balance ability, and the ABC Scale was used to measure balance confidence.

The BESS assessment is a standard assessment which utilized double-leg, single-leg, and tandem stances as the participant stood on either firm or foam surfaces, with eyes closed and hands on their hips. To reduce learning effects, stances were presented in a Latin square sequence. For a total of six trials per condition, during each 20 second trial, the number of deviations from the testing stance was counted as “errors.” Errors included, for example, extending hands and arms away from one's hips in order to maintain their balance, opening one's eyes, stepping or stumbling (losing balance), crouching (hip abduction or flexion beyond 30°) to be able to balance, or remaining out of the proper testing position for >5 seconds. Each error was given a point of 1, and errors were counted throughout each trial. A higher score meant a larger number of deviations and thus was interpreted as lower ability to balance. However, a lower score meant fewer deviations and thus was interpreted as a better ability to balance.

To assess participants' balance confidence before and after training, the ABC Scale was used. The survey questions included how confident one was doing daily activities in or out of their home, for example: “How confident are you that you will not lose your balance or become unsteady when you: Walk around your house? Reach for something above your head? Walk on icy sidewalks?” A score of 100% would mean that one was completely confident that they would not lose their balance during a certain activity. Conversely, a score of 0% would mean that one had absolutely no confidence that they would not lose their balance during that activity. The participants self-reported their balance confidence by providing a number that ranged anywhere from 0 to 100% for each scenario. Each participant was blind to their ABC survey answers from their initial (before training) assessment.

### 2.3. Statistical Analysis

Participants' data were organized, tabulated and postprocessed in Microsoft Excel (Version 16.26). In terms of statistical analysis, for each group, for each test condition, trials were pooled from which means and standard errors were computed for the above parameters. Significant differences were determined by using statistical analysis between the assessments before and after training. Significant differences were observed as *p* values <0.05 and assessed using *t*-tests for equal sample size and unequal variance. In terms of correlation between balance confidence and balance ability (i.e., ABC Scale and BESS error results), each participant's results were plotted (BESS error was the dependent variable, and ABC Scale result was the independent variable). Linear regressions were performed, and R-squared (*R*^2^) values were computed. *R*^2^ is a statistical measure that represents measures the relationship (0 lowest and 1 highest) between the linear regression and the dependent variable.

## 3. Results

### 3.1. Balance Error Scoring System (BESS) Results

**Figure 1:**
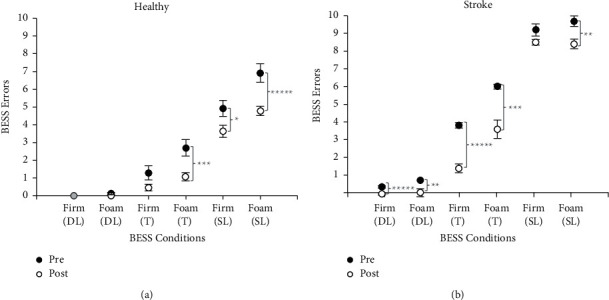
(a) 27 healthy and (b) 7 chronic stroke participants' BESS errors before (filled circle) versus after (open circle) training as a function of BESS test conditions (stances: DL = double leg, *T* = tandem, SL = single leg; support surface: firm or foam); means and standard errors are shown. Significance levels are shown as ^*∗*^*p* < 0.05,  ^*∗∗*^*p* < 0.02,  ^*∗∗∗*^*p* < 0.01,  ^*∗∗∗∗*^*p* < 0.002,  ^*∗∗∗∗∗*^*p* < 0.001.

Balance improvements were observed in both healthy and stroke participants.  Figure 1 displays the BESS errors (before and after) for healthy participants and stroke participants for the various standing conditions. Test conditions from “easiest” to “most difficult” were firm surface/double-leg stance (Firm/DL), foam surface/double-leg stance (Foam/DL), firm surface/tandem stance (Firm/T), foam surface/tandem stance (Foam/T), firm surface/single-leg stance (Firm/SL), and foam surface/single-leg stance (Foam/SL). For all BESS conditions, eyes were closed and thus participants did not have visual information.

For both the healthy and stroke groups, in comparing after training to before training, there were significant decreases (increases in balance ability) in the total number of BESS errors (*t* = −3.55, d*f* = 35, *p* < 0.001 and *t* = −8.20, d*f* = 11, *p* < 0.001, respectively). For the healthy participants, for the easier conditions (Firm/DL and Foam/DL), there were no significant differences before and after. However, differences (decreases) were observed in the most difficult conditions (i.e., Foam/T: *t* = −3.15, d*f* = 39, *p* < 0.01; Firm/SL: *t* = −2.28, d*f* = 48, *p* < 0.05; Foam/SL: *t* = −3.70, d*f* = 37, *p* < 0.001). For the stroke participants, there were significant decreases in several conditions after versus before observed as an increase in balance ability (i.e., Firm/DL: *t* = −5.70, d*f* = 10, *p* < 0.001; Foam/DL: *t* = −3.11, d*f* = 6, *p* < 0.02; Firm/T: *t* = −8.31, d*f* = 10, *p* < 0.001; Foam/T: *t* = −4.59, d*f* = 7, *p* < 0.01; Foam/SL: *t* = −3.15, d*f* = 12, *p* < 0.02). It is of note that their performance improved for the three most difficult standing conditions (i.e., standing on the foam surface).

### 3.2. Activities Specific Balance Confidence (ABC) Results

**Figure 2:**
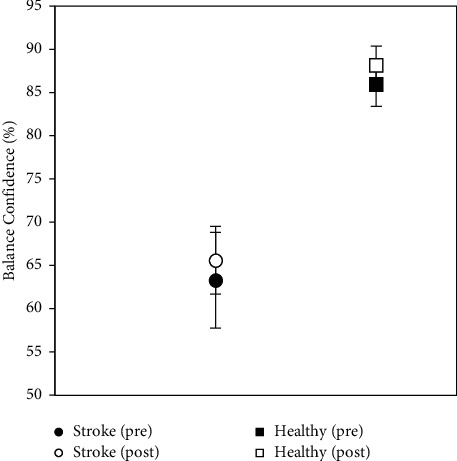
ABC Scale scores for 27 healthy (circle) and 7 chronic stroke (square) participants before (filled) versus after (open) training; means and standard errors are shown.

As expected, balance confidence (ABC Scale) results were significantly different (higher) (before: *t* = 3.72, d*f* = 9, *p* < 0.01; after: *t* = 5.00, d*f* = 10, *p* < 0.001) in the healthy participants compared to the stroke participants (Figure 2). The ABC before training for stroke participants was 63.3% ± 14.6% and for healthy participants was 86.0% ± 13.4%, and the ABC after training for stroke participants was 65.6% ± 10.4% and for healthy participants was 86.9% ± 11.9%. For both healthy and stroke groups, there were no significant differences before versus after training.

### 3.3. Examining the Relationship between Balance Ability and Balance Confidence

**Figure 3:**
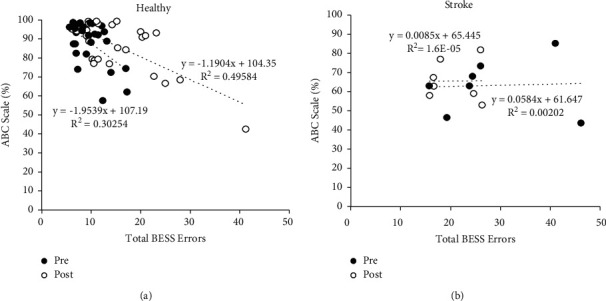
(a) 27 healthy and (b) 7 chronic stroke participants' ABC Scale scores before (open circle) and after (filled circle) training versus total BESS errors; trendline and *R*^2^ values are shown.

Figure 3 shows ABC Scale results as a function of total BESS errors with linear regression and *R*^2^. For the healthy participants, it was observed that before training, there was a negative trend between balance confidence and ability (i.e., an increase in ABC (balance confidence) led to a decrease in BESS errors (or increase in ability to maintain balance)) and *R*^2^ values were higher compared to stroke participants. After training, the relationship between the two measures (slope) steepened (i.e., increases in ABC led to further decreases in BESS errors (or greater increases in maintaining balance) compared to after training). However, for the stroke participants, both before and after training showed no correlation (near zero *R*^2^) between ABC and BESS errors, even though the BESS errors decreased (i.e., balance ability improved) after training.

When observing the trend relating healthy participants' balance performance and their perception of their balance improved after training, it is observed that as BESS errors (balance ability) decreased, this caused an increase in the ABC Scale (balance confidence) as shown in Figure 3(a). However, when observing the trend for the stroke participants (both before and after training), independent of balance ability (as indicated by BESS errors), their balance confidence remained constant and balance ability were independent of one another (uncorrelated), as shown in Figure 3(b).

## 4. Discussion

For older adults, both lack of balance ability and lack of balance confidence are two major challenges which can contribute to falls and activity avoidance. The purpose of this study was to investigate both balance ability and balance performance in older adults and their relationships before and after training. This topic is relevant in that healthy individuals and patients may receive balance training to improve their balance abilities; however, if balance confidence is not improved in parallel, then their capacity to do daily activities still would remain limited.

In both healthy and stroke groups, our results showed that balance ability improved with training as observed in our BESS results. When ABC Scale results were pooled, balance confidence was significantly higher in the healthy group than in the stroke group, as expected. Yet, balance confidence (within each group) did not significantly increase after training compared to before training. In healthy older individuals, it was observed that increases in balance confidence (ABC Scale results) were correlated with increases in balance ability (i.e., greater decreases in BESS errors) after training compared to before training. For the stroke group, there were observed improvements in balance ability (i.e., decreases in BESS errors across nearly all conditions). However, despite improvements in balance ability in this group, balance confidence did not improve. And furthermore, balance confidence and balance ability, before nor after training, were not correlated. Stroke patients vary in the depth of intensity of their disabilities with some impacted more drastically than others. This could explain their low balance confidence even after training. Here, the results showed that the survivors of stroke did not perceive their increased balance ability and therefore their confidence in their abilities did not increase. However, we are conservative in this interpretation due to our small sample size for the survivors of the stroke group. Different from our study and training protocol which involved standing and walking exercises, other previous studies targeting trunk control and core stability in chronic survivors of stroke had led to improvements in balance confidence [30].

As discussed above, we sought to examine the relationship between balance confidence and ability. Previous studies examined FOF, balance confidence (via ABC Scale), and performance. Rosén et al. [31] identified significant positive correlations between falls self-efficacy and clinical measures of balance. Other clinical measures of balance (e.g., walking speed, Berg Balance Scale (BBS), and Timed Up and Go Test) have been related to balance confidence, whereby better balance performance equates to higher balance confidence [32–35]. This is aligned with our finding that improved balance ability (decreased BESS errors) was correlated with an increase in balance confidence (increases in ABC Scale results) in healthy adults; balance performance was related to better balance confidence in healthy individuals both before and after training, with improvements of this relationship after training. However, in individuals several months after stroke, both improvements of balance performance before and after training were not correlated to enhanced balance confidence.

In previous studies, it had been observed that in some cases, loss of balance confidence appears independent of experiencing falling [36]; this suggests that balance confidence could relate to a perception of instability as opposed to actual instability. Older adults who reported lower balance confidence were less willing to attempt faster walking speeds, even though no fall had occurred [32]. In our study, it is a possibility that because of the relatively short training duration, the participants were unable to “test” their balance ability doing various activities denoted in the ABC Scale to observe a change, even though there was an actual change in their abilities as observed by the BESS. In other words, the ABC Scale asks participants to assess confidence in completing activities that individuals may not have attempted since the start of the training (e.g., walking on icy sidewalks). Therefore, it may be challenging for participants to quantify their level of balance confidence accurately if these activities were not attempted within the duration of the study.

The associations between balance confidence, standing balance control and gait, and FOF are lacking for stroke populations and will continue to be a topic for further studies; an understanding of these relationships in older individuals, but particularly in people within the chronic stroke stage (at least 6 months after stroke), is not well understood. One study that sought to identify relationships between balance confidence and gait was [17]. This particular study aimed to quantify relationships between balance confidence and balance and gait features in individuals with chronic stroke. Here, in our study, one possible explanation is that the survivors of stroke did not perceive their increased balance ability after training and thus confidence did not increase. Our survivor of stroke group results may be explained by the notion that self-efficacy plays a role in balance confidence and, more importantly, that balance confidence is connected to with what one thinks (perceives) they can do and perhaps not their actual skill [22]. In order to improve higher fall-risk individual's (e.g., survivors of stroke) balance perception, there may be a need for additional balance confidence training interventions coincident with balance ability training.

For older adults, a consequence of decreased sense of balance ability may lead to individual's reluctance to carry out daily activities, even when they have the capacity to perform these tasks. In a previous study [12, 37], when highly active adults were compared with less-active adults, there was no difference in fall-efficacy scores between groups; however, the highly active group reported less fear of falls. Reducing fall risk (e.g., via training) may not necessarily enhance balance confidence, nor reduce fear of fall. Added interventions, self-efficacy, and balance confidence training may also be needed aside from a focus placed on balance ability.

Recent studies have integrated cognitive behavioral therapy (CBT) alongside balance training as a way to simultaneously promote balance ability and decrease FOF and avoidance behavior [38]. CBT is a psychotherapeutic approach that targets negative cognitive, emotional, or behavioral responses tied to balance confidence and FOF. The combination of CBT with training was observed to promote balance confidence among chronic stroke patients (55–85 years old). Another study by Wetherell et al. [38] utilized an intervention program which targeted cognitive restructuring, exercise, and home safety evaluation towards reducing FOF/avoidance behaviors. A psychologist provided a fall prevention education on reducing personal and environmental risk factors and reducing injury from falls. FOF was measured by the Falls Efficacy Scale (FES) and showed decreases after training. CBT atop balance training may hold potential to improve balance confidence in conjunction with balance ability.

### 4.1. Limitations

Although the results presented here are encouraging, there were some limitations. In terms of ethnicity, most participants were either Caucasian or African American. In terms of gender, in the healthy group, most of the participants were female and in the stroke group, most participants were male. Convenience sampling was used in this study in that, all older adults lived in the Washington, DC Metropolitan area. Furthermore, we had limited access to survivors of stroke; however, as previously stressed, the main focus of this study was on older adults; a larger number of participants were healthy older adults seeking to maintain or improve their balance. The stroke participants had a broad range of time since stroke; however, it was important to include this group to determine baseline results due to the societal relevance. Due to our smaller sample size for the higher fall-risk group (survivors of stroke in the chronic stage), we are conservative in our conclusions although they are encouraging; because of the timing of the COVID-19 pandemic, we were unable to train and assess additional participants who had suffered from stroke. Another limitation was that it was challenging to dictate to and monitor both health and stroke participants towards limiting their activities external to the study. Instead, we requested that they keep their “regular” activities consistent throughout the weeks they were enrolled in the study. Furthermore, in this study, we chose to focus exclusively on standing balance but plans will address gait in future works. Promisingly, these results form the basis for future research.

## 5. Conclusions

Our study sheds new light on the relationship between balance ability and balance confidence in older individuals. In addition, there are also implications for older survivors of stroke in the chronic stage which requires further investigation. Because fall risk, balance confidence, and balance ability all can affect one's ability to mobilize in their daily scenarios, it is important to establish the nature of these relationships. Knowledge of these relationships can determine, as well as sculpt, which interventions are suitable to improve balance confidence balance ability and thus reduce falls in the older population.

## Figures and Tables

**Table 1:** Demographics of survivors of stroke.

Subject	Age	Male/female	Type of stroke/notes	Time elapsed since stroke (years)	Ethnicity	Fall and/or fall-related injury (within past 5 years)	Dizziness or vertigo	Ailments	Activities	Vision
S1	69	Male	Aneurysm of the left internal carotid artery; Subarachnoid hemorrhage; suffered subarachnoid bleeding in the right hemisphere; uses ankle foot orthosis (AFO) and a cane occasionally	33	Caucasian	No	No	No	Walk in mall about 35′, PT work 1 or 2×/month	Glasses

S2	65	Male	Cerebellar stroke; uses cane; however, is able to walk without it and also regularly exercises	3	Caucasian	No	Yes	No	Daily walking	No

S3	61	Male	Weakness on left side due to stroke; uses stimulation as opposed to AFO, also very active with regular exercise	3	Caucasian	Yes	No	Weakness on the left side	Personal trainer, pilates	Glasses

S4	56	Male	Left thalamic intraparenchymal hemorrhage; multiple lacunar infarcts, microhemorrhages, and small vessel disease; does not use cane or walker	1, 3	African American	No	Yes	No	Yes	Glasses

S5	81	Female	Suffered a small acute stroke in the high right frontal lobe with no hemorrhage; may have suffered a second stroke but did not stay for diagnostic; no cane nor walker used	0.75	Caucasian	Yes	Periodic	Feet pronate	Water aerobics	Glasses

S6	72	Male	Suffered a stroke but did not provide doctor's assessment	10	Caucasian	Yes	Did not provide	Did not provide	Did not provide	Glasses

S7	59	Female	Suffered a stroke but did not provide additional information on type of stroke	26	Caucasian	No	No	Hearing loss/vision	OT/PT, treadmill, bike	Glasses

**Table 2:** Demographics of healthy participants.

Subject	Age	Male/female	Ethnicity	Fall and/or fall-related injury (withn past 5 years)	Dizziness or vertigo	Ailments	Activities	Vision
H1	78	Female	Caucasian	Yes	No	Poor dorsiflexion in the left foot	Water aerobics and walking	Glasses
H2	65	Female	Caucasian	No	No	No	No	Glasses
H3	70	Female	Caucasian	No	No	Did not provide	Did not provide	No
H4	70	Female	Caucasian	No	No	Unsure	Visits to wellness center and Jazzercise	Glasses
H5	67	Female	Caucasian	No	No	No	Weight lifting, yoga, and hiking	Glasses (for reading only)
H6	67	Female	Caucasian	No	No	No	Walking and yoga	Glasses
H7	71	Female	Caucasian	Yes	Yes	Hearing loss	Weight-bearing exercises and walking	No
H8	70	Male	Caucasian	Did not provide	Yes	Chronic disk impairment	1×/week with med ex-trainer	Glasses (for reading only)
H9	66	Female	Caucasian	No	No	Uneven leg strength (self-diagnosed)	Working out with a trainer, walking, and yoga	Glasses (for reading only)
H10	72	Female	Caucasian	No	No	No	Yes	Glasses
H11	63	Female	Caucasian	No	No	No	Walking, gardening, and yoga	No
H12	68	Female	African American	No	No	Arthritis	No	Glasses
H13	63	Female	African American	No	No	No	Yes	Glasses (for reading only)
H14	74	Female	Caucasian	No	No	No	Water aerobics	Glasses
H15	80	Female	Caucasian	No	No	No	Water aerobics	Glasses
H16	63	Female	Caucasian	No	No	No	Water aerobics	No
H17	78	Female	Caucasian	No	No	No	No	Glasses
H18	71	Female	European	No	Periodic	No	Walking, jogging, and biking	No
H19	71	Female	Caucasian	No	No	No	Low-impact aerobics and yoga	Glasses
H20	64	Female	African American	No	No	No	Balance and strength exercises	Glasses
H21	62	Male	African American	No	No	No	1-2×/week of exercise	Blind in one eye
H22	83	Female	Caucasian	Yes	No	Arthritis	Water aerobics	No
H23	68	Male	Caucasian	No	No	No	Stretching, stationary bike, and yoga	Glasses
H24	65	Female	African American	Did not provide	Did not provide	Did not provide	Did not provide	Did not provide
H25	67	Female	Caucasian	No	No	No	Strength and flexibility cycling and treadmill walking	No
H26	68	Male	African	Did not provide	Did not provide	Did not provide	Did not provide	Glasses
H27	75	Male	Caucasian	Yes	No	No	Jogging and swimming	Glasses

## Data Availability

Data are not freely available due to ethical concerns such as participant privacy. All presented data were acquired by the Center for Biomechanical and Rehabilitation Engineering (CBRE) laboratory.
